# Formulation development, in vivo bioequivalence and pediatric PBPK modeling studies of taste-masked ciprofloxacin chewable tablets

**DOI:** 10.1038/s41598-023-43423-0

**Published:** 2023-09-26

**Authors:** Muhammad Talha Usmani, Muhammad Harris Shoaib, Fahad Siddiqui, Farrukh Rafiq Ahmed, Rabia Ismail Yousuf, Muhammad Talha Saleem

**Affiliations:** 1 Department of Pharmaceutics, Faculty of Pharmacy and Pharmaceutical Sciences, University of Karachi, Karachi, 75270 Pakistan; 2 Bioavailability and Bioequivalence Research Facility, Faculty of Pharmacy and Pharmaceutical Sciences, University of Karachi, Karachi, 75270 Pakistan

**Keywords:** Paediatrics, Public health, Therapeutics, Computational science

## Abstract

A taste-masked chewable tablet of ciprofloxacin using ion exchange resin Kyron T-134 for enhancing compliance for the paediatric population was developed. The drug-to-resin ratio was optimized for maximum taste masking by studying the effects of soaking time (X1) and mixing time (X2) on complexation (%) using Central Composite Rotatable Design (CCRD). The resin complexes were characterized by bitterness score, DSC, FTIR, and PXRD. The complex was further formulated and optimized into chewable tablets through full factorial design, The optimized formulation was subjected to a bioequivalence study, and a virtual approach of PBPK modelling was adapted to predict the pharmacokinetics of the drug in the paediatric group. The drug resin ratio of 1:1.5 yielded an optimum drug loading of 99.05%. The optimized formulation shows minimum disintegration time with more than 99% drug release within 30 min. The formulation F-9 was found to be bioequivalent with a geometric mean ratio of C_*max*_, T_*max*_, AUC_0–t_, and AUC_*0–∞*_ within 90% CI. It was concluded that quality by design approach can successfully be applied to optimize the drug resin ratio and PBPK modeling is a successful predictive tool for estimating the pharmacokinetics of ciprofloxacin HCl in the paediatric population.

## Introduction

**Figure 1:**
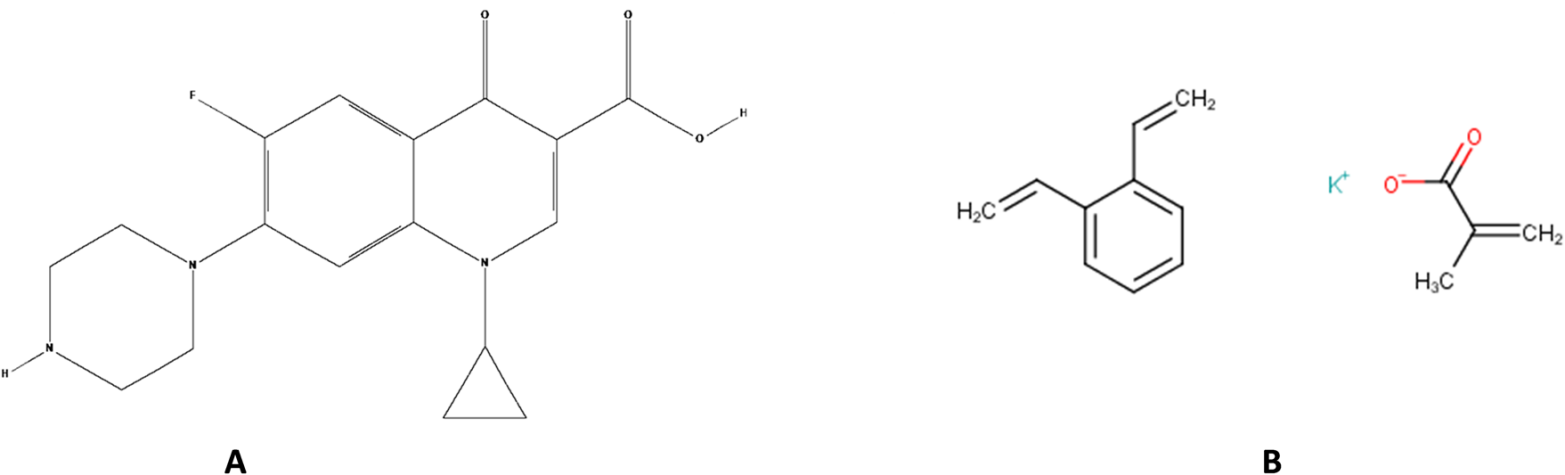
(**A**) Chemical structure of Ciprofloxacin hydrochloride (**B**) Polacrilin potassium.

Taste is an important factor that influences patient compliance with oral dosage forms. It has significant importance, particularly for pediatric patients. An estimation reflects that more than 50% of patients do not take their medications as prescribed if they find it unpleasant to taste^1^. This signifies the need for the masking bitter taste of drugs and for this purpose, numerous techniques have been employed. Among them, ion-exchange resin complexation has been used by several investigators owing to its simplicity, efficiency, and economic viability^2,3^. The technique involves weak binding of the drug molecule to an oppositely charged functional group of the resin forming drug-resin complex which does not dissociate at the salivary pH in the oral cavity. But as soon as it reaches the gastric environment with a relatively low pH, the complex is readily dissociated and releases the drug owing to the abundance of H^+^ ions in the stomach which have more affinity to ion exchange resin than the drug^4–6^. Kyron T-134 is the potassium salt of a low cross-linked carboxylic cation-exchange resin prepared from methacrylic acid and divinylbenzene. It attracts positively charged ions. Ciprofloxacin has a positively charged piperazine functional group through which the negatively charged carboxylic acid group of resin makes complex^7^. Ciprofloxacin (1-cyclopropyl-6-fluoro-1,4-dihydro-4-oxo-7-(1-pipera-zinyl)-3-quinoline carboxylic acid)^8^ (see Fig. 1a,b)^9,10^. It is widely prescribed for a variety of infections in adults, and for inhalation anthrax, complicated urinary tract infections, and pyelonephritis in children^11^. The drug has an extremely bitter taste^12^ and thus, it becomes challenging to formulate a product that should possess a pleasant taste without compromising the bioavailability.

In this study, taste-masked chewable tablets of ciprofloxacin were developed, using complexation with ion exchange resin along with the investigation of the effect of critical factors i.e. soaking time (X_1_) and mixing time (X_2_) on the extent of the ion exchange process. The process was further optimized using the Design of Experiment (DoE) approach during the preparation of the drug resin complex and formulation of chewable tablets^13,14^. To assess the bioequivalence of the optimized formulation, a two-period, randomized crossover bioequivalence study was conducted in healthy volunteers with the immediate release reference product (Ciproxin). The optimized formulation of ciprofloxacin chewable tablet was found to be bioequivalent to the reference product. The mechanistic physiologically based pharmacokinetic (PBPK) modeling was performed to predict drug pharmacokinetics in the pediatric population.

## Methods

### Materials

Ciprofloxacin Hydrochloride was received as a gift sample from Pharmagen Private Limited, Pakistan. Acidic cation exchange resin, Kyron T-134 was a kind gift from Corel Pharma, India. Magnesium stearate (Peter Greven Asia, Malaysia), Croscarmellose Sodium (Gujarat Microwax Private Limited, India), Sodium Lauryl Sulphate (SLS) (Stepan Philippines Quaternaries, Inc., Philippines), Mannitol (Qinqdao Bright Moon Seaweed Group Corp. Ltd, China) and Sucralose (JK Sucralose Inc., China). The chemicals and solvents (HPLC grade) used for the analysis of the drug were purchased from Merck, Darmstadt Germany.

### Ciprofloxacin–resin complexation

The different quantities of resin were transferred to deionized water and allowed to soak for 15 min under stirring. Ciprofloxacin hydrochloride was added separately to each of these resin-containing beakers to obtain different drug-resin ratios (1:1, 1:1.25, 1:1.5, 1:1.75 and 1:2) and stirred using a magnetic stirrer for 30 min at room temperature. The mixtures were then filtered, and residues were washed with 50 ml of deionized water^46^. The unbound drug in the filtrate was estimated as per the assay method recommended in USP-38, to compute the percent complexation of the drug. The High performance liquid chromatography (HPLC) system comprised of the isocratic pump (LC-20 AD, Shimadzu, Kyoto, Japan), a UV–visible detector (SPD-20A, Shimadzu, Kyoto, Japan) auto-sampler (SIL-20 AC, Shimadzu, Kyoto, Japan), a column oven (CTO-20A), and a degasser (DGU-20A5R, Shimadzu LC-20A) using reverse phase C-18 column (Teknokroma, Mediterrania Sea 18, 5 µm, 250 × 4.6 mm i.d.) operated at 40 °C. The mobile phase consisted of 0.025 M phosphoric acid adjusted with triethylamine to pH 3.0 and acetonitrile (87:13) pumped at 1.5 ml/min. The UV detector was set at 278 nm and data were acquired through Lab solution software (version 5.65, Shimadzu, Kyoto, Japan).

Statistically designed experiments having two factors at three levels (− 1, 0, + 1) using Central composite design (Design-Expert 8, version 8.0.7.1, Stat-Ease Inc, Minneapolis, MN, USA) were performed to study the effect of two critical factors—soaking time (X_1_) and mixing time (X_2_) on percent drug complexation. The design led to 13 experiments with 5 replicates as shown in Table 1.

**Table 1:** Trial batches (DRC-1 to DRC-13) of percent ciprofloxacin-resin complexes.

Experiment code	Soaking time (min)	Mixing time (min)	Percent drug complexation (%)
DRC-1	15	30	98.62
DRC-2	5	15	96.21
DRC-3	15	30	98.55
DRC-4	29.14	30	99.07
DRC-5	15	30	98.13
DRC-6	15	8.76	94.8
DRC-7	25	45	99.05
DRC-8	15	51.21	98.72
DRC-9	25	15	97.62
DRC-10	0.85	30	96.9
DRC-11	15	30	98.46
DRC-12	15	30	98.66
DRC-13	5	45	98.41

### Characterization of drug resin complex

The drug resin complex was characterized by instrumental techniques like Fourier Transform Infrared (FTIR) Spectral study, X-ray diffraction analysis, Differential Scanning Calorimetric (DSC), analysis, and in vitro dissolution study of the ciprofloxacin HCl resin complex.

Ciprofloxacin HCl, resin, physical mixture of drug and resin, and optimized drug-resin complex were powdered and transferred into the sampling window, and then their IR spectra (Cary 630, FTIR Spectrophotometer, Agilent, USA) were recorded over the region 4000–650 cm^−17^.

An X-ray diffractometer (Model: JDX-3532, JEOL, Japan) was used to examine the crystallinity of the drug in the complex and/or formation of crystalline substance in the case of drug-resin complex and physical drug-resin mixture. The X-ray copper target tube Kα (λ = 1.5418 Å) was operated at a crystal monochromator voltage of 30 kV and current of 30 mA. The scanning was carried out over the 2θ range of 8° to 60 °C^15^.

Thermal analysis of the components of drug resin complex like a pure drug, Kyron T-134, physical mixture, and DRC was carried out on a Discovery DSC 250 instrument (TA instruments, New Castle, USA). The thermograms were recorded in the temperature range of 40–360 °C for each sample (4–10 mg) in a closed pan^16^.

Dissolution studies of the drug resin complex were performed according to USP-38. Dissolution apparatus II (USP) (Sotax Dissolution Apparatus, Switzerland) was used in which the amount of drug resin complex equivalent to 250 mg of ciprofloxacin was taken in 900 ml of dissolution medium (0.1N HCl). The temperature was maintained at 37 ± 0.5 °C with the paddle rotating at 50 rpm. The samples were taken at 10, 15, 30, 45, and 60 min and analyzed using the HPLC method as mentioned in assay^17^. The samples were injected in triplicate.

### Optimization and pharmaceutical quality evaluation of chewable tablets

The trial batch of the drug-resin complex with optimal complexation and taste was selected for subsequent formulation development of taste-masked ciprofloxacin chewable tablets.

The wet granulation method was used to granulate mannitol for 5 min in a Kenwood planetary mixer (KMC570, China) with the wet slurry of the drug-resin mixture to form a solid wet mass and the obtained granules were dried at 55 ± 5 °C in a fluid bed dryer (Retsch TG-200, Germany) until loss on drying was NMT 5%.

**Figure 2:**
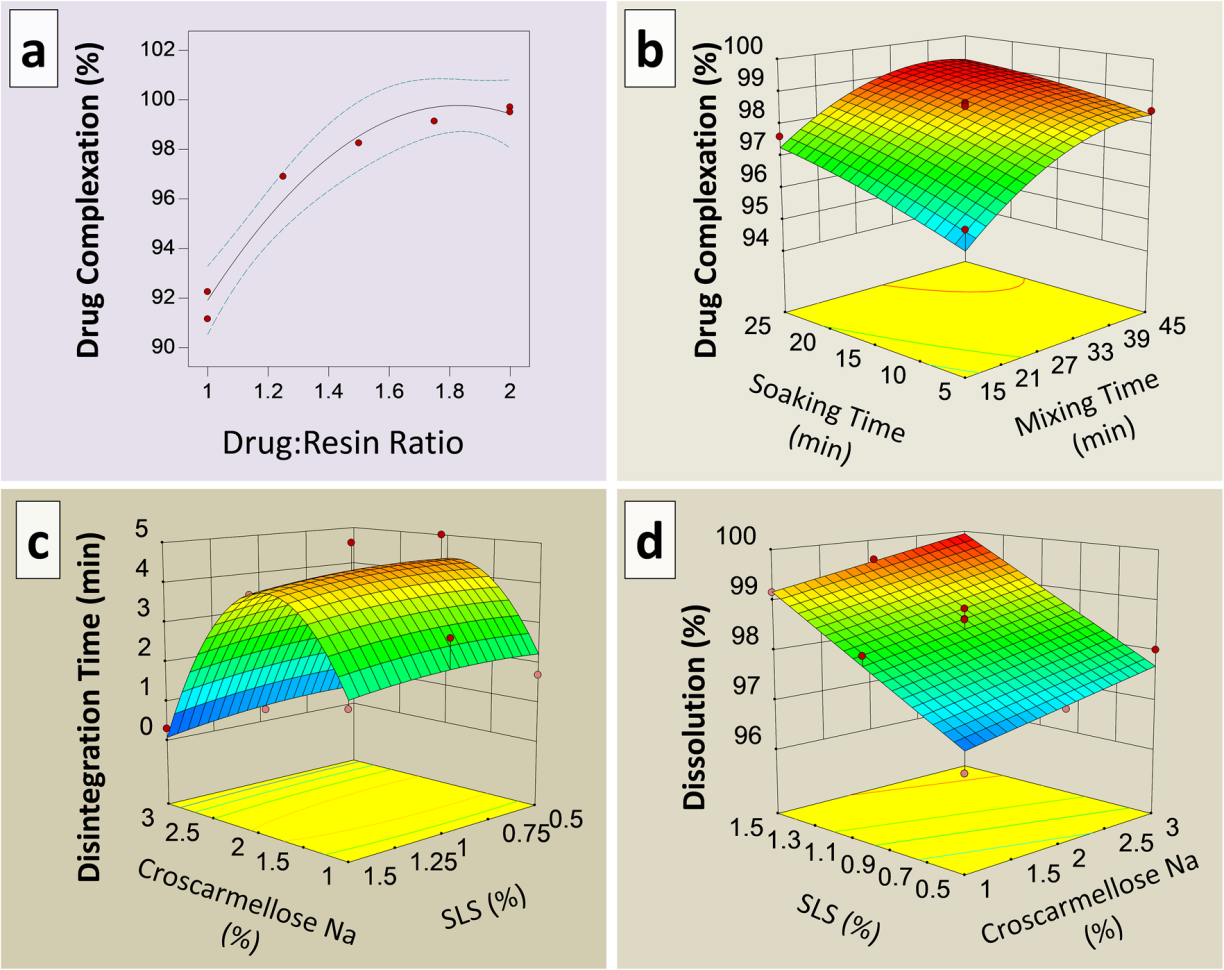
Response surface showing relationship of (**a**) drug:resin ratio and percent complexation. (**b**) Independent variables (soaking and mixing time) and drug complexation. (**c**) Independent variables (Croscarmellose Sodium and SLS) and disintegration time. (**d**) Independent variables (Croscarmellose Sodium and SLS) and dissolution.

The formulation was numerically optimized using Design Expert software (Design-Expert 8, version 8.0.7.1, Stat-Ease Inc, Minneapolis, MN, USA). A three-level factorial design was applied with the disintegrant (croscarmellose sodium) concentration of 1–3% and the solubilizer (SLS) concentration of 0.5–1.5% selected as independent variables, while disintegration and dissolution were used as responses to these variables (Fig. 2a–d). Lots F1–F13 listed in Table 2 were prepared to obtain the formulation with optimal disintegration and dissolution properties.

**Table 2:** Ciprofloxacin 250 mg chewable tablets trial batches (F1-F13).

Material name	F1-1	F-2	F-3	F-4	F-5	F-6	F-7	F-8	F-9	F-10	F-11	F-12	F-13
Ciprofloxacin HCl (mg)	291.100*	291.100	291.100	291.100	291.100	291.100	291.100	291.100	291.100	291.100	291.100	291.100	291.100
Kyron T-134 (mg)	436.500	436.500	436.500	436.500	436.500	436.500	436.500	436.500	436.500	436.500	436.500	436.500	436.500
Sodium Lauryl Sulphate (mg)	9.000	13.500	13.500	4.500	4.500	9.000	4.500	9.000	13.500	9.000	9.000	9.000	9.000
Mannitol (mg)	95.400	99.900	108.900	117.900	99.900	104.400	108.900	113.400	90.900	104.400	104.400	104.400	104.400
Croscarmellose Sodium (mg)	27.000	18.000	9.000	9.000	27.000	18.000	18.000	9.000	27.000	18.000	18.000	18.000	18.000
Sucralose (mg)	9.000	9.000	9.000	9.000	9.000	9.000	9.000	9.000	9.000	9.000	9.000	9.000	9.000
Chocolate Flavour (mg)	20.000	20.000	20.000	20.000	20.000	20.000	20.000	20.000	20.000	20.000	20.000	20.000	20.000
Magnesium Stearate (mg)	12.000	12.000	12.000	12.000	12.000	12.000	12.000	12.000	12.000	12.000	12.000	12.000	12.000
Deionized Water (mL)	QS**	QS	QS	QS	QS	QS	QS	QS	QS	QS	QS	QS	QS
Total (mg)	900.00	900.00	900.00	900.00	900.00	900.00	900.00	900.00	900.00	900.00	900.00	900.00	900.00

*291.10 mg Ciprofloxacin Hydrochloride is equivalent to 250 mg Ciprofloxacin.

**Quantity sufficient.

First, granules obtained by wet granulation and subsequent drying of mannitol with DRC slurry were sieved through sieve 40 and loaded into a 5 l capacity double-cone blender (Changzhou Jiafa Granulating Drying Equipment Co., Ltd, China). Croscarmellose sodium, sucralose, and chocolate flavor were then added by passing through USP sieve no. 40, and mixed for 25 min. Finally, to this blend magnesium stearate was added by passing through USP sieve no. 40, and mixed further for 2 min. The blended granules were then compressed using 19.2 mm × 8.9 mm oblong-shaped punches to obtain tablets of 900 mg weight on a 4-station rotary tablet-making machine (Cadmach Machinery Co. Pvt. Ltd., India).

The formulated chewable tablets were evaluated for various parameters such as weight variation, hardness, friability, percentage drug content (assay), disintegration time, in-vitro dissolution, and taste evaluation.

The USP weight variation test was performed by weighing 20 tablets individually on a weighing balance (AUW 220, Shimadzu, Kyoto, Japan), calculating the average weight, and comparing the individual tablet weights to the average. The tablets pass the USP test if no more than 2 tablets are outside the percentage limit and if no tablet differs by more than twice the percentage limit. In this case, the weight variation limit was ± 5% taking into account the tablet weight of 900 mg^17^.

The hardness of the tablets was evaluated using a Sotax Multitest 50 hardness tester (Sotax, Aesch BL, Switzerland). 20 tablets were subjected to a hardness test and the average was calculated.

Friability was determined using Pharmatest friabilator PTF 100 (Pharmatest, Hainburg, Germany) which consisted of a circular plastic chamber having a single drum. Ten tablets were weighed and placed in the apparatus which was rotated at 25 rpm for 4 min. After the revolution, the tablets were de-dusted and weighed. The friability is given by the formula:

1 $$F= \left(1-\frac{{W}_{o}}{W}\right)\times 100,$$

where W_o_ is the weight of the tablets before the test and W is the weight of the tablets after the test. The weight loss should not be more than one percent^17^.

The drug content was determined by transferring 20 finely powdered tablets (equivalent to 250 mg of ciprofloxacin) to a 100 ml volumetric flask. To this solution, 2 ml of 5N HCl was then added and the volume was made with distilled water. The volumetric flask was stirred in a sonicator for 15 min with intermittent shaking. The samples were appropriately diluted in the mobile phase and filtered and analyzed by HPLC technique that was used in drug loading studies. The test was carried out in triplicate^17^.

The disintegration time of the tablets from each formulation was determined using a USP basket rack assembly (Pharma Test PTZ-S, Hainburg, Germany). The in vitro disintegration test was carried out at 37 ± 2 °C in 900 ml of distilled water. The prescribed limit for disintegration of chewable tablets is NMT 15 min according to the international pharmacopoeia^18^.

Dissolution of tablets was performed according to USP Apparatus II (Sotax Dissolution Apparatus, Switzerland). Compressed tablets of optimized formulation were placed in 900 ml of dissolution medium (0.01 N HCl) as used in studying the dissolution rate of resin complexes. The temperature and speed of the apparatus were maintained at 37 ± 0.5 °C and 50 rpm, respectively. The test was carried out on six tablets and aliquots were taken at time intervals of 10, 15, 30, 45, and 60 min, filtered with Whatman # 41 filter paper, and analyzed at 278 nm by HPLC as per the assay method mentioned in USP^17^.

The bitterness evaluation test for ciprofloxacin chewable tablets was performed on healthy human volunteers as per the method described by Kawano et al.^19^. The study was conducted under the guidance of the Declaration of Helsinki, Good Clinical Practice (GCP) guidelines, and after getting approval from the independent institutional bioethics committee of the University of Karachi (IBCPH 10). The signed written informed consent form was taken from each volunteer and the test was performed by a group of six human volunteers previously trained in taste evaluation. The volunteers rinsed their mouths thoroughly before and after tasting the tablets The subjects chewed the tablets and held them in their mouths for 30 s and then spit them out. Based on taste, numerical scoring was done by each volunteer according to the following scale: 0-No bitterness, 1-Slight bitterness, 2-Moderate bitterness, 3-Strong bitterness.

### Comparative dissolution testing

To evaluate the in-vitro performance of the optimized ciprofloxacin chewable tablets (F-9) compared to an established brand i.e. Ciproxin 250 mg tablet, comparative dissolution testing was performed according to WHO guidelines^20^. The WHO recommended media were selected that cover the physiological pH range i.e., 0.1 N Hydrochloric acid (pH 1.2), pH 4.5 acetate, and pH 6.8 phosphate buffer solution.

The dissolution profile of 12 units each of the test (ciprofloxacin 250 mg chewable tablets) and reference (Ciproxin 250 mg tablet) was determined in each dissolution medium. Taking the mean dissolution values of each time point, the similarity factor (*f*_2_) was determined.

Within 15 min of operation, if dissolution from both reference and test products was > 85%, the dissolution patterns were concluded similar, and therefore no calculation of *f*_2_ is required.

Any value of *f*_2_ above 50 is acceptable. The higher the *f*_2_, the greater the similarity between the two products, which proves the equality of the product and the similarity in performance^21^.

### Stability study

The optimized formulation (F-9) was tested for stability (Biobase BJPX-MT250, Biobase, Shandong, China) under accelerated conditions (40 °C/75% RH). The tablets were individually packed in aluminum blisters in unprinted unit cartons and kept under the conditions specified above for 6 months. The test were carried out after three and six months of stability, evaluating the tablets for appearance, hardness, disintegration time, active ingredient content, in vitro dissolution and taste^22^.

### Bioequivalence study of ciprofloxacin test and reference formulations

The optimized taste-masked formulation of ciprofloxacin was selected as a test product compared to the innovator brand Ciproxin 250 mg tablets as a reference product for conducting a single-center, two treatment cross-over bioequivalence study in healthy male volunteers.

The study was conducted under the guidance of the Declaration of Helsinki, Good Clinical Practice (GCP) guidelines, and after approval from the independent Institutional Bioethics Committee of the University of Karachi (IBCPH 10)^23^. The volunteers were provided with detailed information about the aim, methodology, and possible associated risks of the current study in oral and written form in both English and native language (Urdu). Written signed informed consent was taken from the participants prior to the study, and they were advised to report any adverse reactions during the study period.

Healthy male volunteers were selected to minimize inter-subject variability. The age of the enrolled subjects was between 21 and 33 years and their body weight was between 64 and 81 kg. Medical history and demographic data were collected from each volunteer followed by physical examination (weight, height, blood pressure, temperature and pulse rate), blood biochemistry, renal function, urinalysis and ECG before the study. Any subject with clinical laboratory results outside the standard range was excluded from the study. Alcoholics, tobacco, or nicotine users of any kind were also not included in the study. Volunteers were not allowed to take any over-the-counter medications and/or multivitamin/mineral supplements for two weeks before and during the study. Caffeinated beverages and grapefruit juice were also prohibited during the study period. Subjects with a history of participation in any drug investigational study or had donated blood within the last 4 months were also excluded.

The current study was of a single-centre, single-dose, open-label, randomized, two treatments, two period crossover design. The treatments were administered after randomization to 12 subjects in either 1 or 2 sequences, with half of them receiving the test and the other half receiving the reference product, separated by a washout period of 7 days^24^. Vital signs were monitored before drug administration, during and at the end of each tolerability assessment period^25^. Blood samples (5 ml) were collected in vacutainers through an IV canula at 0, 0.5, 1, 1.5, 2, 2.5, 3, 4, 6, 8, 12, and 16 h. Plasma was separated by centrifuging the sample at 4000 rpm for 5 min and the separated plasma was immediately stored at − 20 °C until analysis^25^.

Plasma concentrations of ciprofloxacin were analyzed using a previously published laboratory validated HPLC–UV-based method using simple protein precipitation as sample pre-treatment technique^26^. Non-compartmental analysis of pharmacokinetic parameters was performed using Kinetica software (version 5.1, Thermoelectron corp., USA).

Two-way Latin square ANOVA was applied to log-transformed data of pharmacokinetic parameters obtained from a randomized crossover design to evaluate formulation, period, sequence, and subject (within the sequence) effects with a significance level of 0.05 (α)^27^. A two-one-sided *t*-test was used to determine whether the average values of pharmacokinetic (PK) parameters assessment data obtained after administering the test and reference products were comparable. A 90% confidence interval was calculated for the ratio of the average PK measures for the test and reference products. The ratio of geometric mean and 90% confidence interval was calculated using Kinetica software. Bioequivalence was concluded if the resulting 90% CIs were within the pre-specified limit according to FDA guidance i.e. 0.80–1.25 or 80–125% for the ratio of the averages^27^.

### Physiologically based pharmacokinetic (PBPK) modeling

The physiologically based pharmacokinetic (PBPK) model based on the Advanced Compartmental Absorption and Transit (ACAT) model for the taste-masked chewable tablet of ciprofloxacin was simulated using GastroPlus 9.8.3 (Simulations Plus, Inc., Lancaster, CA). The approach was to develop and establish absorption models in pediatric populations for oral chewable tablets formulated from drug-resin complexes developed from Kyron T-135. Various experimental and literature-extracted input parametric values are incorporated as physicochemical, physiological, and pharmacokinetic properties and expressed in Table 3^28–36^. Initial validation was performed by comparing simulated Intravenous (IV) bolus profile of ciprofloxacin with the adult in vivo experimental data. In addition, a comparison of in vivo adult oral data with the simulated plasma profile for IR chewable tablets was also performed. Simulations were performed in both the fasted and fed states to compare the effects of food on the extent of intrinsic absorption of ciprofloxacin from the gut wall. The obtained results were then implemented to correlate pediatric plasma profiles with the adult real-time experimental in vivo data. Therefore, three databases were established: for the IV bolus 250 mg, chewable IR tablet 250 mg (fasted condition), and chewable IR tablet 250 mg (fed condition). Pediatric physiology was generated by the Gastroplus PBPKplus internal Population Estimates for Age-Related (PEAR) Physiology module. Before developing the correlation, the adult plasma concentration–time profile of the optimized formulation was analyzed using the PKPlus module of the software and various compartmental parameters were exported to the pharmacokinetic tab of the main software of GastroPlus. From the dissolution results obtained at different pH media, simulations were also carried out for different physiological pH values. The relative accuracy between predicted PK parameters and observed PK parameters was calculated by comparing the fold error (FE) between the observed and predicted values according to formula (see Eq. 2)^37^. The simulated adult fed and fasted PK parameters were compared with those calculated from the current bioequivalence study of ciprofloxacin HCl,, whereas, the PK data reported by Peltola et al. was used to calculate the FE from paediatric simulated data^38^.

**Table 3:** Physicochemical, physiological and pharmacokinetic input parameters used for the development of PBPK model of ciprofloxacin using GastroPlusTM.

Input parameter	Value	Source
*Log P*	1.7	Olivera et al.^36^
*pKa*	6.2/8.59	Olivera et al.^36^
Molecular weight (*MW*) (g/mol)	385.8	Balbas-Martinez et al.^30^
Aqueous solubility (*S*) (mg/ml)	10 to 30	Olivera et al.^36^
Jejunal effective permeability (*P*_*eff*_) (cm/sec × 10–4)	0.29	Wang et al.^28^
Unbound percent in human plasma (% F_up_)	0.60–0.80	Wingender et al.^32^
Human blood to plasma concentration ratio (R_bp_)	1.25	ADMET Predictor™
Cl total (l/h/kg)	0.34	Gonzalez et al.^34^
Cl Renal (l/h)	22	Höffken et al.^31^
Cl biliary (ml/min/kg)	1.25	Lettieri et al.^33^
T_*1/2*_ (h)	4.78	PKPlus™
K_12_ (1/h)	2.11	PKPlus™
K_21_ (1/h)	1.598	PKPlus™
Vss (l/kg)	1.74–5	Vance-Bryan et al.^35^
Vd (l/kg)	1.1	Montgomery et al.^29^

2 $$\mathrm{FE }= Predicted\;value / Observed\; value.$$

The FE value of less than 2 exhibits similarity with the reported PK parameters^39^.

## Results

### Ciprofloxacin–resin complexation

The percent drug complexation values for the drug-to-resin ratios of 1:1 (in duplicate), 1:1.25, 1:1.5, 1:1.75, and 1:2.0 (in duplicate) were 91.77 (n = 2), 96.90, 98.25, 99.13 and 99.60% (n = 2), respectively (Table 1). Since the percentage increase in the active ingredient complex was not significant from 1:1.5, increasing the amount of resin for a slight increase in the active ingredient loading did not make economic sense. Therefore, a drug: resin ratio of 1:1.5, was selected for further investigation^40^. The relationship of drug-to-resin ratio is shown in Fig. 2a.

### Optimization and characterization of drug resin complex

A central-composite rotatable design (CCRD) with two factors at three levels was chosen as the experimental design. The key factors studied were soaking time and mixing time at three levels (− 1, 0, and + 1) and thirteen formulations (DRC1–DRC13) of the drug resin complex were prepared at a ratio of 1:1.5 as shown in Table 1 and Fig. 2a,b. At the highest values of both input variables, a complexation of 99.05% was achieved.

### Fourier transform infrared (FTIR) analysis of ciprofloxacin and drug resin complex

**Figure 3:**
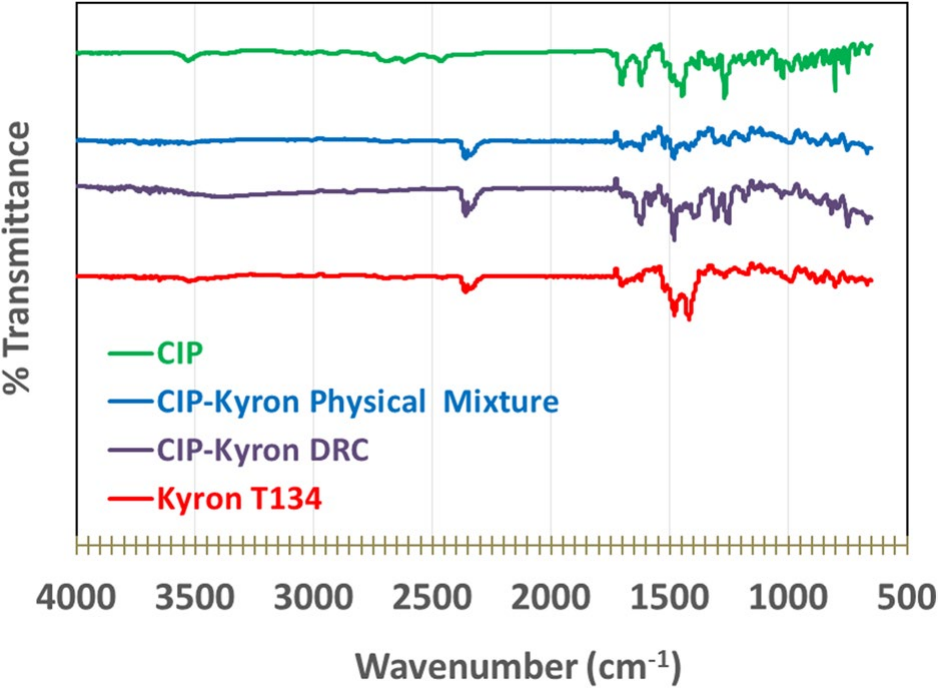
FTIR spectra of (**a**). Ciprofloxacin (API) (**b**) Kyron T-134 (**c**) Ciprofloxacin–Kyron physical mixture and (**d**) Ciprofloxacin–Kyron complex.

FTIR Spectra as shown in Fig. 3 were used to examine any interaction between ciprofloxacin and Kyron T-134. The spectrum of pure drug showed a characteristic NH stretching peak at around 3400 cm^−1^. The spectrum of the drug resin complex depicted the absence of the previously observed peaks in the region 3500–2800 cm^−1^ which was different from that of the physical mixture and the pure drug showing the transformation of the drug into a complex.

**Figure 4:**
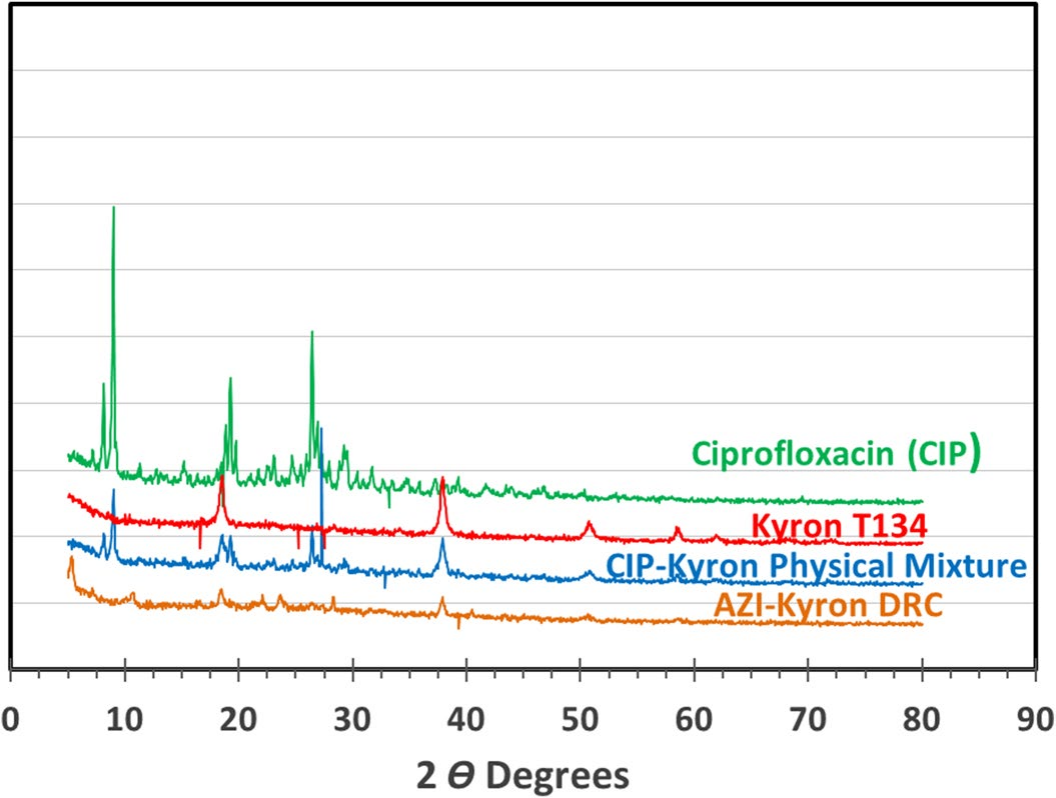
X-ray diffractograms of (**a**). Ciprofloxacin (API) (**b**) Kyron T-134 (**c**) Ciprofloxacin–Kyron physical mixture and (**d**) Ciprofloxacin–Kyron complex.

The powder X-ray diffraction pattern of ciprofloxacin, Kyron T-134, the physical mixture of ciprofloxacin and Kyron T-134, and the optimized drug resin complex is shown in Fig. 4. The result of X-ray diffraction showed that the pure drug showed crystalline properties with multiple sharp peaks while Kyron T-134 showed an amorphous structure with diffuse peaks. A physical mixture of ciprofloxacin with Kyron T-134 showed that ciprofloxacin retained its crystalline properties. This further indicates that the drug did not undergo any physical change or penetrate into the resins, while the drug resin complex did not show sharp or significant peak, thus revealing an amorphous nature of the complex.

**Figure 5:**
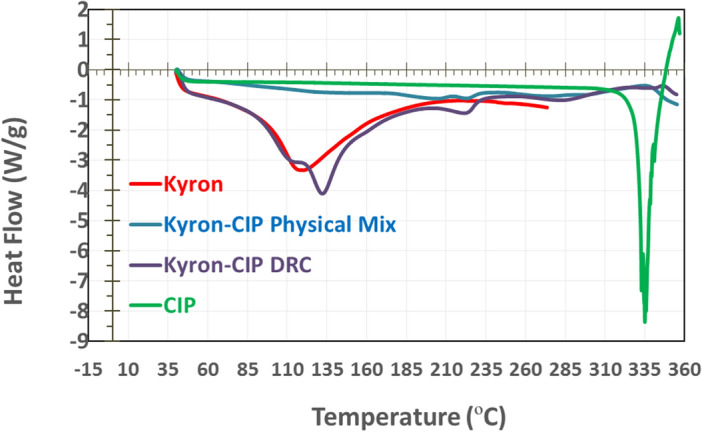
DSC thermograms of individual components of drug resin complex and optimized complex.

Differential scanning calorimetric (DSC) analysis of pure ciprofloxacin HCl revealed an endothermic melting point at approximately 334 °C (see Fig. 5). As observed in the case of the physical mixture the drug and resin, there was no sharp melting peak at any of the two temperature points showing the change in the melting behaviour of the components of the mixture. On the other hand, the drug resin complex also showed a significant change in melting behaviour. The resin shows a shifted melting peak at 135 °C while the drug shows a small exothermic curve at 335 °C, possibly indicating the gradual decomposition of DRC^15^.

### Optimization and pharmaceutical quality evaluation of chewable tablets

The response surface diagram of the experimental design showed an insignificant influence (p-value 0.424) of SLS on disintegration in the ANOVA analysis. However, it was found to have a significant impact on drug dissolution (p-value 0.0056).

The effect of croscarmellose sodium on the disintegration time and dissolution of ciprofloxacin was observed in the range of 1–3% of the tablet weight. The concentration of croscarmellose sodium was found to have a significant impact on the disintegration of tablets. Formulations F-1, F-5, and F-9 contained 3.0% croscarmellose; Tablets of said batches had disintegration times of 0.37, 0.43, and 0.65 min, respectively. Formulation F-3, F-4, and F-8 contained 1.0% croscarmellose sodium and exhibited disintegration times of 4.9, 4.7, and 3.2 min, respectively.

The response surface diagram of the experimental design using ANOVA analysis showed a significant impact of croscarmellose sodium (p-value < 0.00001) on tablet disintegration. However, it has an insignificant impact (p-value 0.942) on the dissolution of the product (Fig. 2c,d).

The results indicated that a value of 0.827 is desirable, with concentrations of 3.0% for croscarmellose sodium and 1.5% for sodium lauryl sulfate considered appropriate to achieve rapid disintegration and dissolution.

After compression, the chewable tablets were evaluated for various pharmaceutical quality parameters like weight variation, hardness, friability, assay content, disintegration time, and in vitro drug release. The results are shown in Table 4. The amount of drug in assay was 98.6–100.04% of the lab in the pharmacopeia i.e. NLT 80% within 30 min^40^. In the bitterness assessment study of all six volunteers, the optimized ciprofloxacin chewable tablets (F-9) were assigned a value of 0, meaning no bitterness is present.

**Table 4:** Pharmaceutical quality evaluation of Ciprofloxacin 250 mg chewable tablet formulations (F1–F13).

Formulations	Quality Control Tests					
	Weight variation (mg) (mean ± SD)	Hardness (N) (mean ± SD)	Friability (%)	Disintegration time (min)	Assay (%) (mean ± SD)	Dissolution (%) (mean ± SD)
F-1	899 ± 2.00	90 ± 7.45	0.41	0.37	99.84 ± 0.29	98.44 ± 0.45
F-2	900 ± 2.62	104 ± 4.65	0.35	4.41	99.65 ± 0.39	99.36 ± 0.20
F-3	901 ± 2.95	111 ± 6.01	0.25	4.85	100.04 ± 0.32	99.86 ± 0.08
F-4	901 ± 2.80	106 ± 6.61	0.21	4.72	99.63 ± 0.07	98.32 ± 0.47
F-5	900 ± 2.42	89 ± 6.68	0.43	0.43	99.46 ± 0.94	98.45 ± 0.44
F-6	899 ± 2.02	105 ± 5.56	0.38	4.20	99.90 ± 0.11	98.66 ± 0.35
F-7	901 ± 2.52	105 ± 5.37	0.32	4.00	99.00 ± 0.14	98.60 ± 0.12
F-8	899 ± 2.37	113 ± 8.08	0.32	3.75	99.43 ± 0.01	98.07 ± 0.54
F-9	900 ± 2.36	97 ± 10.66	0.43	0.65	99.61 ± 0.33	99.30 ± 0.44
F1-0	902 ± 2.97	102 ± 5.15	0.28	3.40	99.53 ± 0.34	98.28 ± 0.60
F-11	900 ± 2.43	105 ± 5.05	0.37	3.25	99.24 ± 0.56	98.56 ± 0.86
F-12	899 ± 2.31	103 ± 5.26	0.36	4.00	99.79 ± 0.17	98.90 ± 0.31
F-13	900 ± 2.50	103 ± 6.08	0.33	3.50	98.69 ± 0.19	97.95 ± 0.69

### Comparative dissolution testing

**Figure 6:**
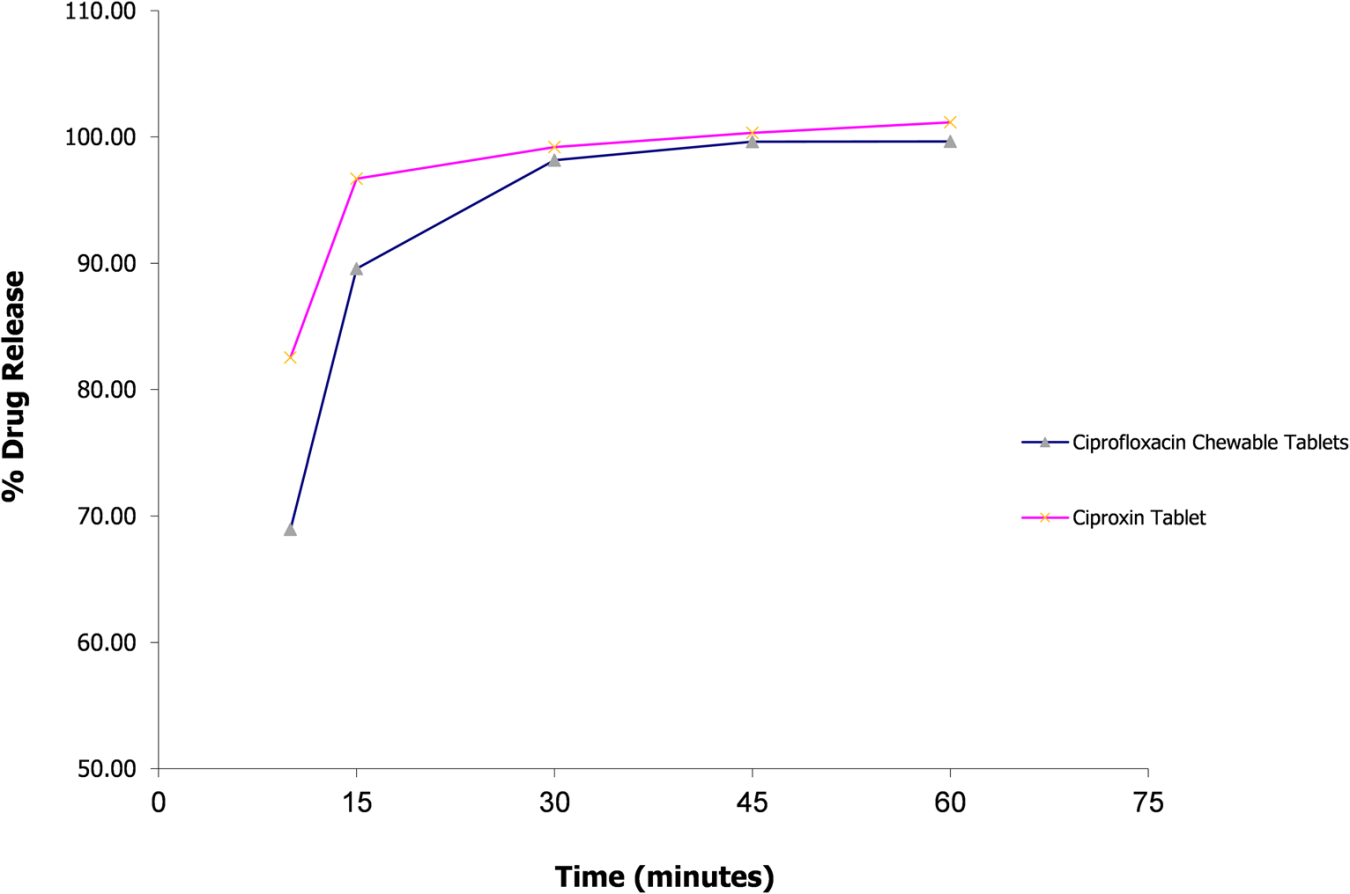
Comparative dissolution profile of Ciprofloxacin chewable tablets and Ciproxin tablets in 0.1N HCl.

**Figure 7:**
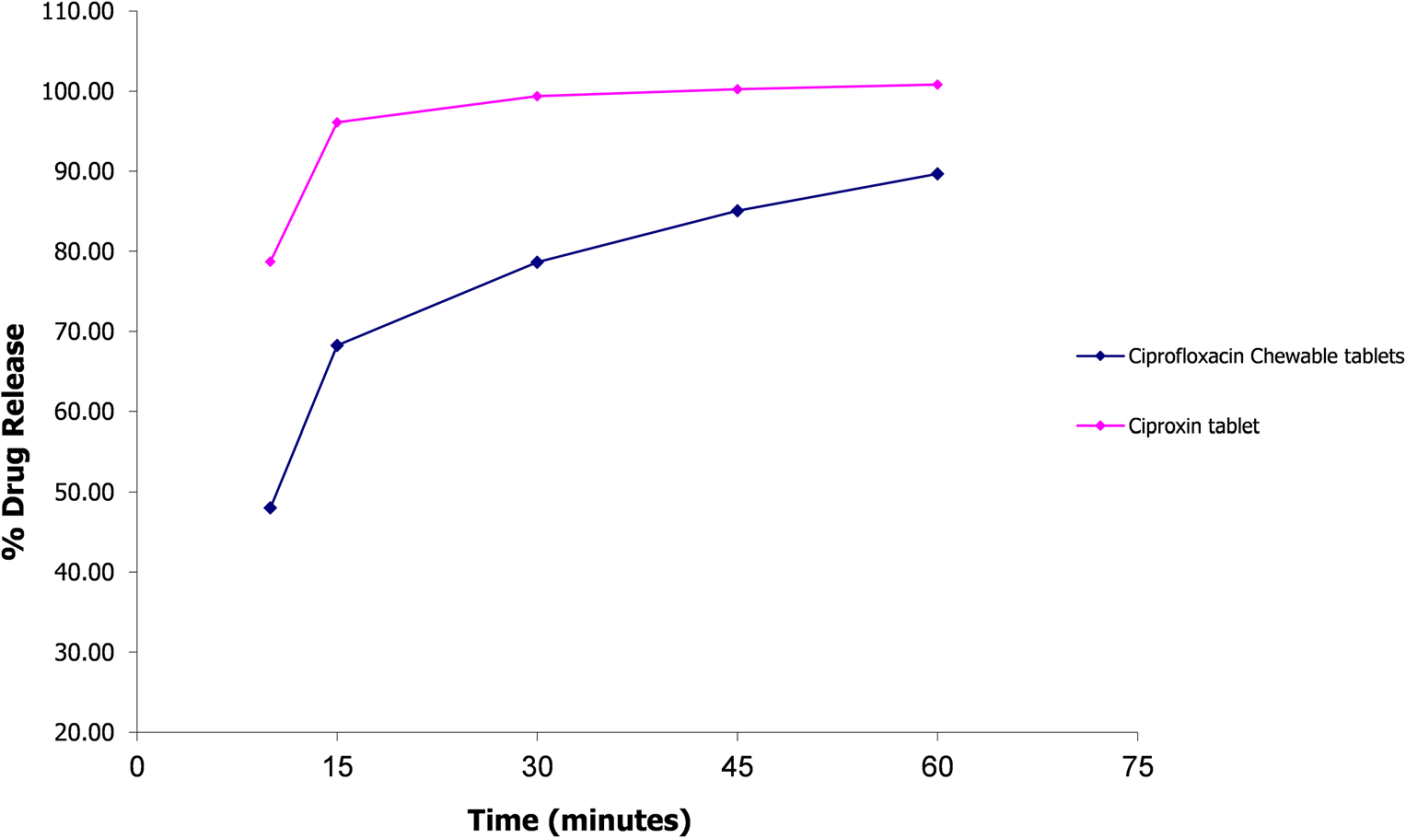
Comparative dissolution profile of Ciprofloxacin chewable tablets and Ciproxin tablets in Acetate Buffer pH 4.5.

**Figure 8:**
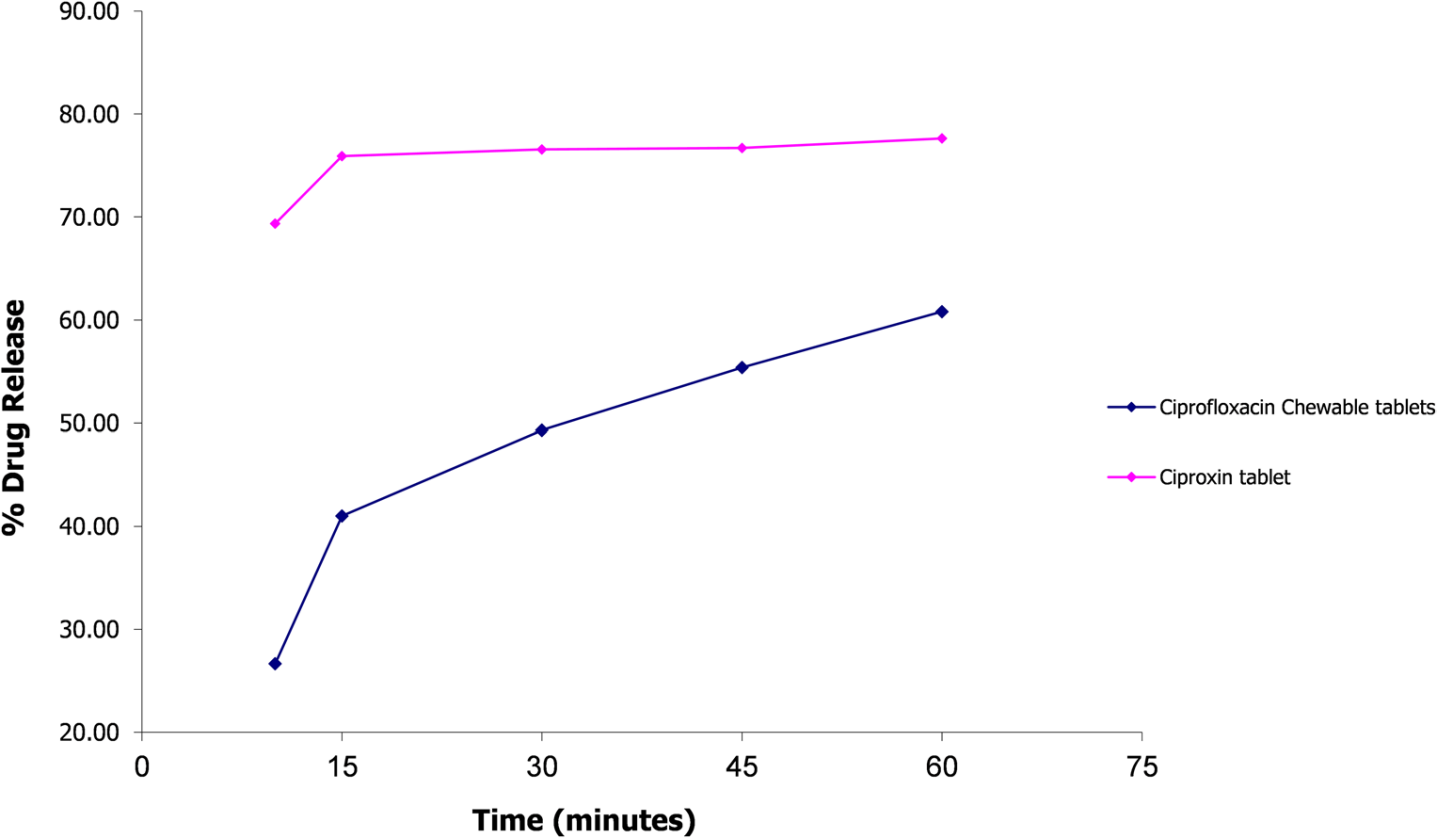
Comparative dissolution profile of Ciprofloxacin chewable tablets and Ciproxin tablets in Phosphate buffer pH 6.8.

According to the guidelines, *f*_2_ calculation is not required if the product/formulation releases more than 85% of the active ingredient within 15 min. Furthermore, drugs that release so much active ingredient in 15 min are unlikely to cause bioavailability problems^21^. Both products i.e., ciprofloxacin chewable 250 mg tablets and Ciproxin 250 mg tablet released more than 85% ciprofloxacin in acidic media i.e., 0.1N HCl (pH 1.2) within 15 min. Therefore, the *f*_2_ test was not applied. Whereas in acetate and phosphate buffers (pH 4.5 and 6.8), *f*_2_ values were calculated to be 32.6 and 26.45 respectively (see Figs. 6, 7, 8).

In the present study, Ciproxin tablets released 77.62% of the drug within 60 min whereas ciprofloxacin chewable tablets released only 60.81% at pH 6.8. It was also found that in acetate buffer (pH 4.5) was higher i.e., 100.82% and 89.67% respectively.

### Stability studies

The tablets were found to be stable under the accelerated temperature and humidity conditions after 6 months. The pharmaceutical quality attributes were found to be acceptable at different time intervals and no significant change in the stability of formulations was observed. Assay and dissolution results was above 99% at the 3rd and 6th month's stability as shown in Table 5.

**Table 5:** Stability study of optimized oral 250 mg Ciprofloxacin chewable tablet formulation (F-9).

Evaluation parameters	Initial	3rd Month	6th Month
Appearance	Complies	Complies	Complies
Drug content (%)	99.61	99.21	99.18
Disintegration time (min)	0.65	0.45	0.40
Hardness (N)	79–110	76–94	68–78
Dissolution after 30 min (%)	99.30	99.42	98.66
Taste	No bitterness	No bitterness	No bitterness

### Bioequivalence study of ciprofloxacin test and reference formulations

The optimized chewable tablet formulation (F-9) was administered for bioequivalence study due to its optimal pharmaceutical properties. Both formulations (reference and test) were well tolerated by all volunteers without any adverse event.

The comparative plasma time concentration profile of reference and test ciprofloxacin tablets is illustrated in Fig. 8. The mean C_*max*_ of the ciprofloxacin chewable tablet was 1.149 ± 0.155 µg/ml, and 1.176 ± 0.226 µg/ml for the reference tablet in fasted state. The time to reach the maximum plasma concentration (T_*max*_) was 1.208 ± 0.257 h, and 1.29 ± 0.257 h for the test and the reference product. The mean AUC_*0–t*_ and AUC_*0–∞*_ values were 5.651 ± 0.779 mg/l × h and 6.036 ± 0.836 mg/l × h respectively for the reference while for the test product the AUC_*0–t*_ and AUC_*0–∞*_ values were 5.012 ± 0.510 mg/l × h and 5.325 ± 0.554 mg/l × h.

The bioequivalence study of the optimized formulation of taste-masked ciprofloxacin 250 mg chewable tablets was conducted in comparison with Ciproxin 250 mg tablets (reference) in a single dose. Statistical analysis of the log-transformed data of the pharmacokinetic parameters using Latin square ANOVA and a two-one-sided *t-*test revealed insignificant sequence, subject, and period effects. The geometric mean ratio of C_*max*_ for reference and test products was 0.9178 with a 90% CI of 0.866–0.973 which is within the confidence limit of bioequivalence (0.85–1.25) while the ratio of T_*max*_ was 1.021 with a 90% CI was 0.837–1.163. When determining the Geometric mean ratio (GMR) for AUC_*0–t*_ and AUC_0–∞_ with 90% CI the values were 0.8905 (90% CI 0.842–0.941) and 0.885 (90% CI 0.835–0.938) respectively. Thus, the respective geometric mean ratios (90% CI) were found within the pre-specified limit i.e. 0.8–1.25 (Table 6) which formed the basis for the conclusion that the formulation is bioequivalent to the reference product.

**Table 6:** Geometric Mean Ratios and 90% CI of log transformed pharmacokinetic parameters of Ciprofloxacin chewable tablets 250 mg (test) vs Ciproxin 250 mg tablets (Reference) (log transformed).

PK parameters	Ratio T/R	90% CI
C_*max*_	0.918	0.866–0.973
T_*max*_	1.021	0.837–1.163
AUC_0*–t*_	0.890	0.843–0.941
AUC_*0–∞*_	0.885	0.835–0.938

The geometric mean ratio of C_*max*_ for reference and test products was 0.9178 with a 90% CI of 0.866–0.973 which is within the confidence limit of bioequivalence (0.85–1.25) while the ratio for T_max_ was 1.021 with 90% CI of 0.837–1.163. When determining the Geometric mean ratio (GMR) for AUC_*0–t*_ and AUC_0-∞_ with 90% CI, the values were 0.8905 (90% CI 0.842–0.941) and 0.885 (90% CI 0.835–0.938), respectively.

### Physiologically based pharmacokinetic (PBPK) modeling

**Figure 9:**
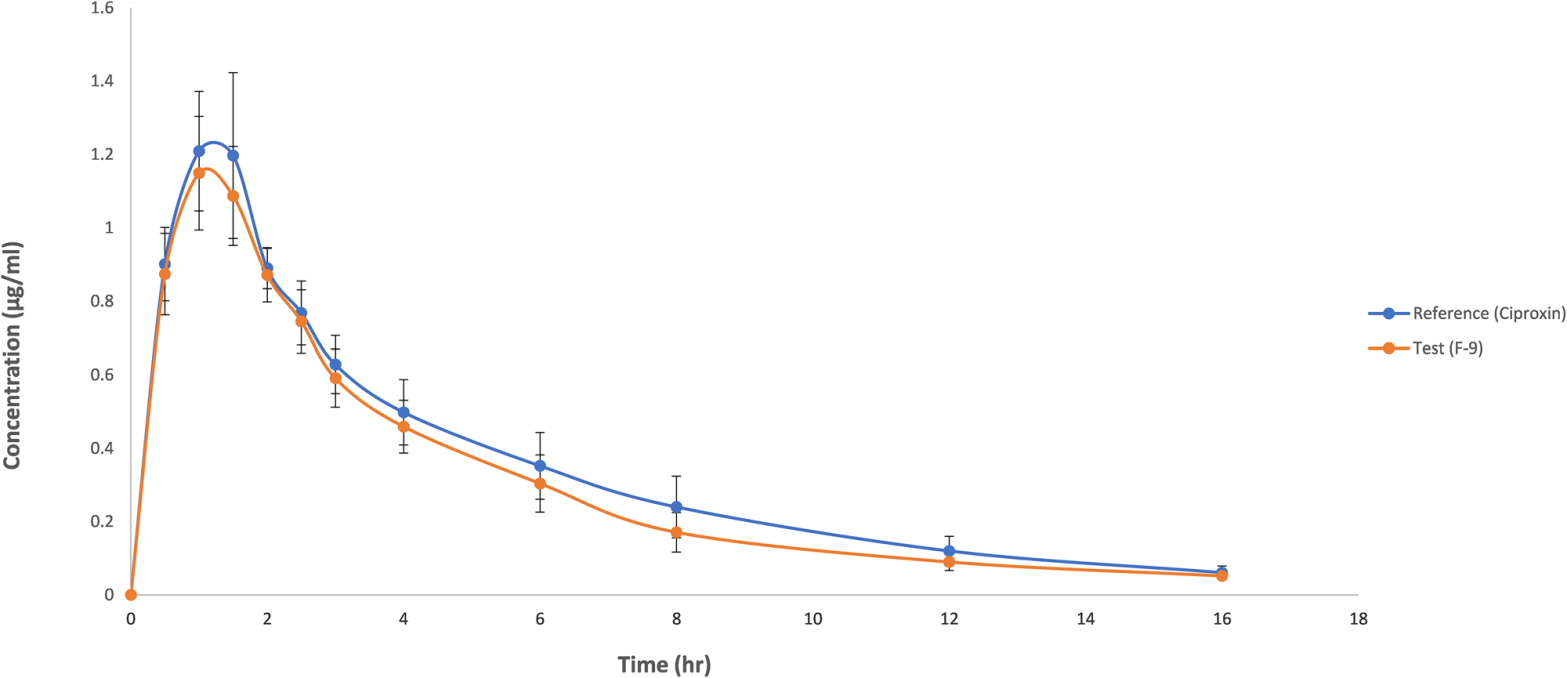
Mean ± SD plot of plasma concentration versus time profile comparison of Ciprofloxacin reference product (Ciproxin 250 mg) and Ciprofloxacin chewable tablets (Test F9) under fasted condition.

The simulation results of the predicted plasma profiles for a 250 mg intravenous bolus in adult humans were performed via the ACAT model. The combined plot of the predicted and experimental plasma profiles showed overlapping curves as shown in Fig. 9a. A simulation for 250 mg oral chewable tablets was performed in both fasted and fed states. showed very little difference in C_*max*_ and T_*max*_ as shown in Fig. 9b,c^41^. Figure 9d–f shows the comparison of the observed adult plasma profile with different simulated profiles for children’s obtained at different dissolution pH values. The comparative pharmacokinetic data of adults under fed and fasted states and, in paediatric group is given Table 7. The fold error (FE) value less than 2 demonstrate similarity between the PK parameters simulated from the in vitro dissolution data of the optimized formulation, (F-9) and those obtained from the in vivo bioequivalence study. The simulated PK parameters of F-9 in the paediatric group also have FE values below 2.

**Table 7:** Experimental and predicted values of pharmacokinetic parameters for oral chewable tablets of ciprofloxacin.

Pharmacokinetic parameters	IV bolus			IR chewable tablets adults Fasted State			
	Observed^31^	Simulated	FE	Observed	Simulated	FE	
C_max_ (mcg/ml)	5.7909	7.748	1.34	1.149	1.0624	0.92	
T_max_ (h)	0.0825	0.072	0.87	1.01	1.6533	1.64	
AUC_0–inf_ (mcg-h/ml)	8.8293	10.462	1.18	5.4589	7.5131	1.38	
AUC_0–t_ (mcg-h/ml)	8.0045	8.9597	1.12	5.0798	6.746	1.33	

Pharmacokinetic parameters	IR chewable tablets adults Fed State			IR chewable tablets Paeds			
	Observed	Simulated	FE	Observed^38^	pH 1.2 (FE)	pH 4.5 (FE)	pH 6.8 (FE)
C_max_ (mcg/ml)	1.165	0.89971	0.77	2.10	2.008 (1.04)	1.9263 (1.09)	1.7394 (1.207)
T_max_ (h)	1.5	2.5067	1.67	1.00	2.1333 (0.468)	2.08 (0.481)	1.9733 (0.507)
AUC_0–inf_ (mcg-h/ml)	5.745	7.5968	1.32	5.30	17.151 (0.309)	16.076 (0.329)	13.709 (0.387)
AUC_0–t_ (mcg-h/ml)	5.3605	6.7379	1.26	–	14.329	13.434	11.466

## Discussion

The present study reports the taste masking of ciprofloxacin using weakly acidic cation exchange resin for the complexation of the drug. From the different grades of cation exchange resins, Kyron T-134, a weakly acidic polyacrylic copolymer was selected as the cation exchange resin for the taste masking of ciprofloxacin hydrochloride. Kyron T-134 uses the hydrogen ion as an exchange ion that helps in the adsorption of the drug. Upon contact with an acidic gastric pH, desorption of the drug occurs due to the high affinity of the resin for the hydrogen ion^42,43^.

When studying the optimal drug to resin ratio, the values ​​of the percentage of complexed drug show an increasing trend with increasing resin content, which is due to the to the increased interaction between the drug molecules and the resin particles^44^. Multiple linear regression analysis (MLRA) was performed to determine the extent of the contribution of different factors over the percentage drug complex^45^. It was observed that the percent drug complex was maximum at a high level of both the soaking time (X1) i.e. 25 min and mixing time (X2) i.e. 45 min. It shows that high levels of mixing and soaking time is desirable for maximum binding of the drug to the resin (Fig. 1b).

The possible mechanism behind complex formation is the adsorption of resin to the drug via ion exchange i.e. the –NH group of ciprofloxacin interacts with the functional group –COOH of Kyron T-134 to form complex^46^. The results are in agreement with the findings of Pisal et al. where they performed complexation with indion-234 resin^7,46^. Instrumental analysis of the drug-resin complex by FTIR showed the conversion of a drug into a complex, evident by the absence of the same characteristic drug peaks in the 3500–2800 cm^−1^ region. Due to the absence of characteristic peaks, an amorphous form is observed after undergoing the DRC preparation process. Differential scanning calorimetric (DSC) analysis of pure ciprofloxacin HCl in Fig. 4 shows endothermic melting at approximately 334 °C, as previously published studies by Pisal et al.^47^ while another study reported a range of 314–323 °C^48^. The pure resin shows a melting curve at around 110 °C as reported in the literature^49^.

A three-level full factorial design was chosen to optimize the ciprofloxacin chewable tablet formulation. Through this design, the effect of sodium lauryl sulfate and croscarmellose sodium on tablet disintegration and dissolution was evaluated. Sodium lauryl sulfate is often used to improve the dissolution of drugs with low solubility. Kimaro et al. used SLS to improve drug dissolution from Albendazole chewable tablets^50^. In the present study, the selected range for sodium lauryl sulfate was 0.5–1.5% of tablet weight. Three studies contained 1.5% SLS and all resulted in > 99% drug dissolution (Table 4). With chewable tablets, there is always a possibility that an intact tablet will be swallowed. Therefore, a disintegrating agent was added to the formulation to disintegrate the tablet within 15 min like conventional uncoated tablets, as recommended by the International Pharmacopoeia^18^. The chewable tablets were found to comply with the uniformity of weight variation test as per USP (900 mg ± 5.0%). The hardness was found to be > 70.0 N for all batches, which is sufficient to withstand mechanical shocks during handling in production and transport. Becasue 100% complexation is not achieved, a small amount of ciprofloxacin HCl was left unbound imparting a slight bitterness. Therefore, sweetener and flavouring agent were added to the formulation to overcome the bitterness of free ciprofloxacin HCl.

Ciprofloxacin has pH-dependent solubility and exhibits a ‘‘U’’ shaped pH-solubility profile. Its solubility is high at pH values below 5 and above 10 while the solubility is minimal at neutral pH, near the isoelectric point. Oishi et al., reported that the solubility of ciprofloxacin is limited at pH 6.8 (< 0.02 mg/ml)^51^. The similarity of dissolution of profile in an acidic medium and dissimilarity in acetate and phosphate media is due to the fact that the ciprofloxacin–resin complex breaks quickly at acidic pH while it takes a longer period to dissociate at higher pH. Though some investigators have conducted routine bioequivalence studies of ciprofloxacin tablets like Azhar et al.^24^ and Abul Kalam et al.^52^ the current study is the first to focus on the bioequivalence of a chewable tablets compared to immediate-release ciprofloxacin tablets. Abul Kalam et al. conducted a bioequivalence study of ciprofloxacin on 24 healthy male volunteers and reported the C_*max*_ of test and reference formulations to be 1.49 ± 0.085 µg/ml and 1.46 ± 0.03 µg/ml respectively, T_*max*_ 1.0 ± 0.27 h and 1.2 ± 0.27 h for test and reference formulations. The AUC_*0–t*_ values were 5.82 ± 0.38 and 5.79 ± 0.67 mg/L × h for reference and test products while the AUC_*0–∞*_ values for test and reference products were 6.86 ± 0.93 and 6.92 ± 0.92 mg/L × h^52^. In another study, Azhar et al. conducted a bioequivalence trial of ciprofloxacin immediate release tablets (250 mg) in a healthy Pakistani population and reported C_*max*_ 1.018 ± 0.018 µg/ml for reference and 1.013 ± 0.006 µg/ml for test formulation. The T_*max*_ observed was 1.109 ± 0.019 h for reference and 1.115 ± 0.006 h for test while AUC_*0-t*_ values of 6.58 ± 0.33 µg/ml × h and 6.63 ± 0.34 µg/ml × h for reference and test products, respectively. The pharmacokinetic properties of the currently proposed formulation were therefore found in agreement with the previously published studies^24^.

**Figure 10:**
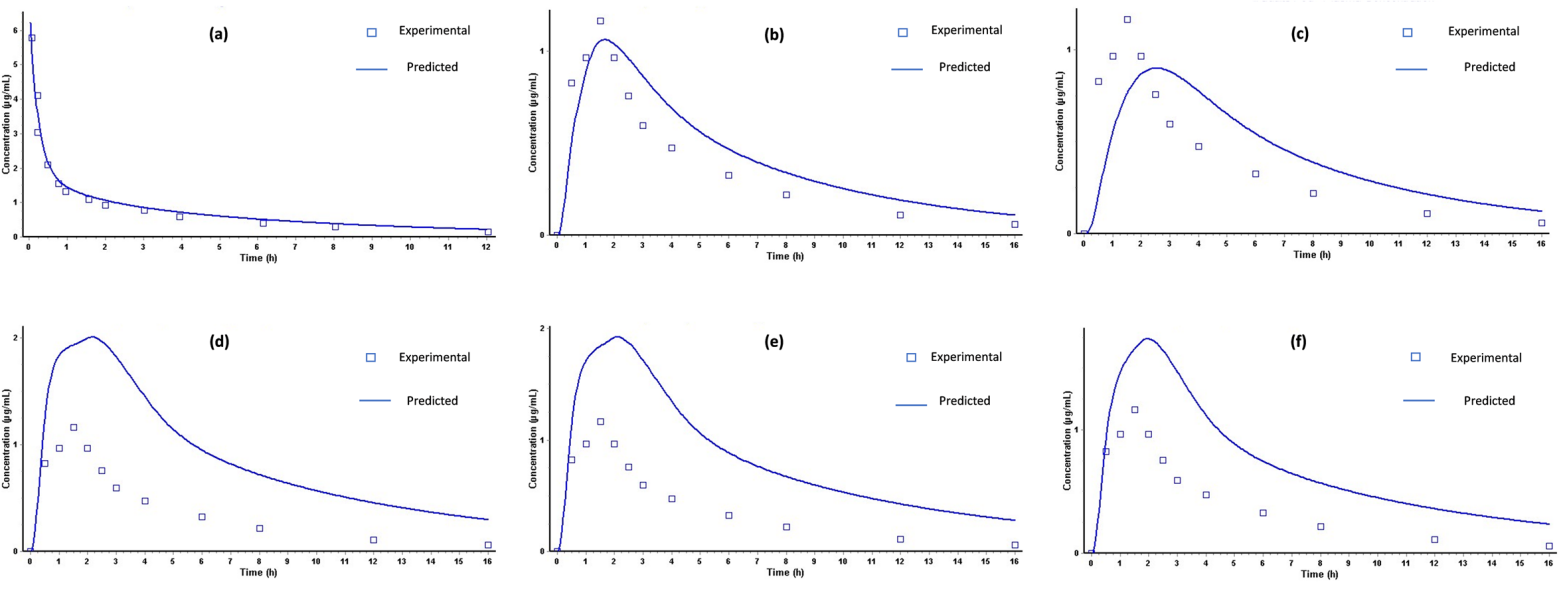
Comparison of observed and predictive Time plasma concentration of Ciprofloxacin 250 mg in (**a**) IV bolus adults (**b**) oral in adults in fasted conditions (**c**) oral in adults in fed conditions (**d**) oral for paediatric in pH 1.2 (**e**) oral for paediatric in pH 4.5 (**f**) oral for paediatric in pH 6.8.

The fold error values below 2 between experimental/observed and simulated results suggest that the predicted values are in good agreement (Table 7). The compartmental analysis of in vivo experimental profiles was then performed by the PKPlus module of GastroPlus to deduce the disposition and elimination of pharmacokinetic parameters as illustrated in Table 3. The comparison of simulated adult profiles with the observed ones and the paediatric (predicted) PK profiles with the adults in fasted sate is presented in Fig. 10a–f. The simulated PK curve (adults) shows no difference in C_*max*_, and AUC values in both fed and fasted conditions, however, T_max_ was found little delayed, i.e., 1.6 h (fasted) and 2.5 h (fed). The apparent increase in the pharmacokinetic parameters of ciprofloxacin in the paediatric population (Fig. 10d–f) is well understood by the fact that the clearance rate of ciprofloxacin is lower in children compared to adults^2^. Since the in vitro dissolution data suggests that the total drug release was reduced by approximately 40% at pH 6.8, the identical behaviour was translated into predicted plasma profiles. When compared with the reported study the FE values of the predicted PK parameters of paediatric group were within the recommended range (< 2), but there was a significant rise in AUC_0–inf_ (17.151 µg × h/mL) and T_max_ (2.11 h)_,_ which may be associated with inter_-_subject variability in pharmacokinetics (see Table 7)^38^. Taste masking has enormous implications for the development of chewable tablets for bitter drug molecules. In the present work, taste masking of ciprofloxacin was effectively achieved by ion-exchange complexation using resin. Formulation development data showed that soaking and mixing times play a critical role in the ion exchange process. The degree of complexation was found to be directly proportional to the soaking and mixing time. FTIR and XRD studies showed the formation of complexes, while the taste evaluation study further confirmed the drug-resin complexation.

The chewable tablets demonstrated a similar release profile to the conventional immediate release tablet of ciprofloxacin while their good taste makes them suitable for administration to paediatric patients. The pharmacokinetics of the taste-masked ciprofloxacin chewable tablets were found similar to that of the reference product in the fed state in terms of C_*max*_, T_*max*_, AUC_*0–t,*_ and AUC_*0–∞*_ and were therefore found to be bioequivalent to the reference product.

This formulation therefore proved to be a viable alternative to conventional oral therapy of ciprofloxacin tablets and suspension.

## Data Availability

The datasets used and analyzed during the current study available from the corresponding author on reasonable request.
